# A review and integration of models on delusion maintenance

**DOI:** 10.1017/S0033291726103705

**Published:** 2026-05-05

**Authors:** Tania M. Lincoln, Henning Romberg, David Torrents-Rodas, Antonia Bott

**Affiliations:** Clinical Psychology and Psychotherapy, Universität Hamburg, Hamburg, Germany

**Keywords:** associative and operant learning, Bayesian, belief revision, belief updating, delusion persistence, etiology, models, paranoia, resistance to change, systematized delusions

## Abstract

Contemporary definitions of delusions highlight their resistance to conflicting evidence as the core feature. However, most etiological models of delusions have focused on delusion formation rather than maintenance and we lack a coherent understanding of why delusions persist. We conducted a systematic literature search of models on delusion maintenance, extracted their core postulates, point to explanatory gaps, and derive an integrated framework. We identified 74 published accounts that include postulated mechanisms of delusion maintenance. We classified the models into six core perspectives that informed them: Bayesian inference (17 models), associative learning theory (6 models), neurobiological (11 models), cognitive–behavioral (23 models), motivational (7 models), and social (6 models). Most models highlight a mechanistic role of avoidance and operant learning, converging on the idea that a delusional explanation is reinforced. Another repeatedly suggested mechanism is that the delusional belief, once formed, influences the way further information is processed. In addition, most models propose a key role of individual deficits and biases. The proposed factors can be combined in temporal progression, including early risk factors and resulting vulnerability, the common proposed mechanism of formation (i.e. search for explanation of ambiguous experiences), and the short- and long-term consequences of the delusional explanation along with feedback loops. By considering numerous factors and their interactions, the integrative model provides a considerably more compelling account of why delusions persist than any single perspective alone. It can help to identify novel directions for research and intervention, such as addressing short-term benefits of delusion maintenance.

## Introduction

Delusions, a core symptom of psychotic disorders, are defined in the Diagnostic and Statistical Manual of Mental Disorders (APA, 2022) as ‘fixed beliefs that are not amenable to change in light of conflicting evidence’ (page 87). Delusions can pertain to any subject, but tend to revolve around fundamental human fears or desires, such as trust, self-worth, love, religion, guilt, health, or death. Paranoid delusions are the most common (Collin, Rowse, Martinez, & Bentall, 2023; Pappa et al., 2025).

At first glance, the seemingly most striking feature about delusions is the fact that they are at odds with the evidence. Upon further reflection, however, forming a belief that is at odds with the evidence as such is nothing unusual. We are all prone to judgment errors. We apply rough heuristics and seldom hesitate for long before forming an opinion (Furnham & Marks, 2013; Kruglanski et al., 2018) which renders our judgments susceptible to error. History offers numerous examples of erroneous beliefs, ranging from the world being a disc to conspiracy beliefs. Even the type of belief typically encountered in people with delusions is not unusual. Delusional ideas, though not directly shared with others, tend to touch on central human themes, such as trust, abilities, or religion and are also endorsed by mentally healthy people. In epidemiological delusion research, this has been particularly well demonstrated for paranoid beliefs (Bebbington et al., 2013; Neidhart, Mohnke, Vogel, & Walter, 2024), but even beliefs in supernatural agencies are widespread across human cultures (Exline & Wilt, 2023). Thus, coming up with an erroneous explanation for an unexpected event is not per se unusual. However, erroneous beliefs, even idiosyncratic ones, may be less clinically relevant if they are revised in response to conflicting evidence. From this perspective, another, and perhaps even more relevant, aspect of the delusional belief is that a person sticks with it against all odds.

When it comes to understanding and successfully treating delusions, the primary challenge is therefore perhaps not only to understand what has caused the beliefs to arise, but also to understand the factors that prevent individuals from revising them. So far, the main focus of theories of delusions has been on explaining belief formation (Denecke et al., 2024). There has been considerably less effort to understand why delusional beliefs are maintained, often over years (Appelbaum, Robbins, & Vesselinov, 2004; Grunfeld et al., 2024), and why even explicit confrontation with contradictory evidence seldom leads to belief revision. Considerations on delusion maintenance are often integrated in a nonprominent manner in the numerous models of delusion formation. This makes it challenging to keep track of the full range of proposed factors and mechanisms. Also, different ideas on delusion maintenance stem from different perspectives (i.e. neurobiological, cognitive, psychodynamic, etc.) with little interdisciplinary discourse. It remains to be determined whether the models overlap, contradict each other, or propose complementary mechanisms, leading to a higher explanatory value in combination.

In this review, we summarize existing explanations on delusion formation and maintenance extracting their core factors. We then integrate the postulated factors into an overarching model and point to explanatory gaps. We conclude with a summary, raise open questions, and derive implications for further research and intervention.

## Models of delusion maintenance

### Identification of relevant models and methods of extraction

**Figure 1.**
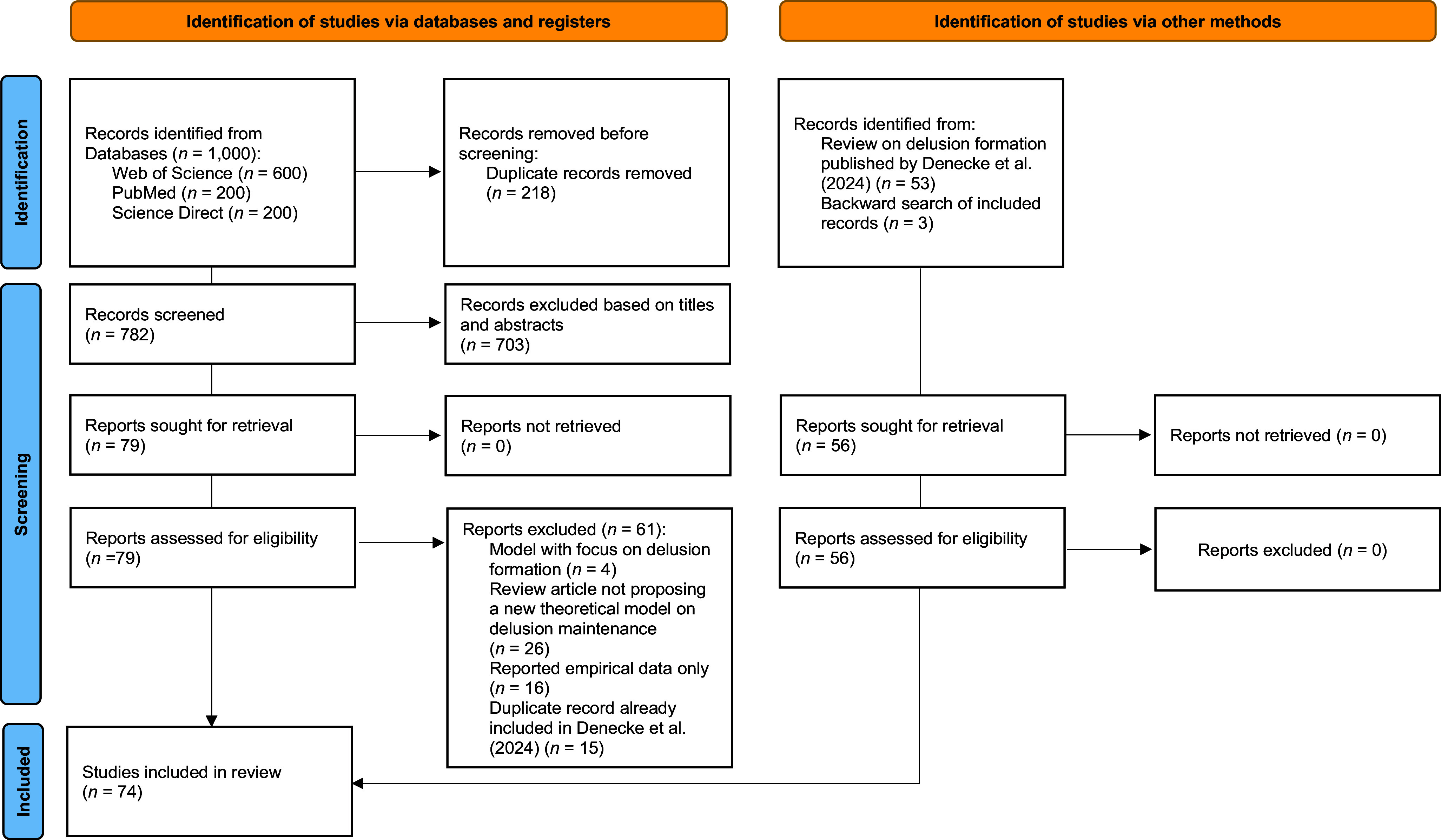
PRISMA flow chart of the literature search on models of delusion maintenance. *Note*: Flow chart adapted from Page et al. (2021). Search terms included keywords related to delusions (i.e. delusion*, deluded, OR), AND maintenance (i.e. maintenance /maintain, stick*, persist*, stability/stable, preserve*, protect*, rigid*; OR), AND theoretical models (i.e. model/models, theory/theories, account, framework, perspective; OR). Further, reference lists of relevant publications were screened for possible cross-reference of additional models. Finally, we screened the models identified in a previous search on delusion etiology (Denecke et al., 2024) for additional models. From the pool of identified studies, we selected the top-ranked 1000 records by screening 600 records from Web of Science and supplementing them with records from the Science Direct and PubMed databases (each *n* = 200).

To obtain an overview of existing models on delusion persistence and extract their main postulates, we conducted a literature search of 1656 peer-reviewed articles in December 2024 and performed an update of this search in January 2026. Articles that were peer-reviewed and explicitly described an original model of delusion maintenance or included hypotheses on delusion maintenance in a more general model of delusions were included (see Supplementary Table). We excluded publications that focused exclusively on general psychosis (e.g., schizophrenia). For details on study selection, see Figure 1.

In total, we identified 74 relevant models. Of these, only two focused on delusion maintenance specifically (Corlett, Krystal, Taylor, & Fletcher, 2009; Westermann, Gantenbein, Caspar, & Cavelti, 2018). The remaining focused on belief formation while adding accounts on belief maintenance. We classified models into the core perspectives that informed them: Bayesian inference (18 models), associative learning theory (6 models), neurobiological (11 models), cognitive–behavioral (25 models), motivational (8 models), and social (6 models). Given the relevance of the models’ postulates to both delusion formation and maintenance and the difficulty of understanding postulates outside of their context, we briefly reiterate the core elements of the respective perspective and their core idea(s) on delusion formation *(highlighted in italics)* before laying out their hypotheses on belief maintenance (**highlighted in bold**). Although the brevity and focus on core postulates come at the cost of not conveying the full complexity of – and the overlap between – models or reiterating all factors they list, we deem this approach sufficient to convey the basic assumptions necessary for extracting the main factors and mechanisms of interest.

### Description of models

#### Bayesian inference informed perspectives

Bayesian inference models (Friston, 2008; Rao & Ballard, 1999) of brain function assume that the brain actively generates probabilistic predictions (i.e. prior beliefs) of its sensory inputs to infer their (probable) causes. Mismatches between these predictions and actual inputs (prediction errors) are used as ‘learning signals’ to gradually refine the internal model of the world, thereby improving subsequent predictions and reducing prediction errors. A central assumption is that the higher the relative reliability (termed ‘precision’) assigned to the sensory inputs compared to the prior belief, the more the prior belief is updated. The internal model of the world is assumed to be organized as a cortical belief hierarchy, with the bottom levels representing beliefs related to the more concrete features of the environment and increasingly abstract beliefs represented at higher hierarchical levels. Within this hierarchy, top-down prior beliefs are fed forward and integrated with bottom-up prediction errors signaled from the next lower cortical level (Friston, 2008).

*Delusion formation.* Drawing on this model of brain function, delusions are proposed to arise from a deviation from optimal Bayesian belief updating (Hemsley & Garety, 1986). In more recent predictive coding models (Adams et al., 2013; Harding et al., 2024; Sterzer et al., 2018), this deviation has been characterized as *aberrant precision weighting*, that is, assigning too little precision to the prior belief or too much precision to the sensory input, or a combination of both. This is proposed to lead to *aberrant prediction error signaling* (Corlett, Honey, & Fletcher, 2016; Fineberg & Corlett, 2016; Fletcher & Frith, 2009) that manifests as feelings of surprise and uncertainty that are resolved by adopting a higher-order delusional belief (Adams et al., 2013; Feeney, Groman, Taylor, & Corlett, 2017; Griffin & Fletcher, 2017; Miyazono & McKay, 2019; Pfuhl, 2017; Sterzer et al., 2018).

*Delusion maintenance.* Several ideas have been put forward to reconcile the paradox of fixed and stable delusional beliefs with the notion of a decreased prior belief precision. One is to differentiate mechanisms for the ‘formation phase’, characterized by an uncertain model about states in the world, from a ‘consolidation phase’, characterized by a **strong impact of the adopted ‘extraordinary higher-order belief’** (Diaconescu, Hauke, & Borgwardt, 2019). Another is to assume a disbalance in the precision weighting of prior beliefs at different positions in the hierarchy. The basic idea here is a reduced weighting of prior beliefs at low hierarchical levels that is compensated by **increased weighting of prior beliefs at high hierarchical levels** (Sterzer et al., 2018). These higher-order beliefs can include delusional beliefs which then ‘sculpt perceptual processing into conformity with delusions and foster their resistance to contradictory evidence’ (Petrovic & Sterzer, 2023, p. 1425). Relatedly, Ashinoff, Singletary, Baker, and Horga (2022) propose overweighted high-level priors to induce a **primacy bias in belief updating**, such that early interpretations dominate subsequent evidence and beliefs become increasingly resistant to change over time. In a similar vein, Fineberg and Corlett (2016) propose delusions to protect the belief system from further destabilization under persistent aberrant prediction error signaling by preventing escalating uncertainty.

#### Associative learning informed perspectives

The focus of associative learning has been on how animals, including humans, adapt to changes in the environment. A fundamental assumption is that they learn to anticipate and respond to significant events by associating them with cues that reliably predict their occurrence. Once such an association is formed, the presence of the cue activates the mental representation of the expected outcome, which initiates an appropriate response (Pearce & Bouton, 2001). Of particular relevance here are the following three concepts: (1) classical conditioning – after pairing a neutral stimulus with an unconditioned stimulus (US) for a number of trials, the neutral stimulus comes to elicit a conditioned response in anticipation of the US, thereby becoming a conditioned stimulus (CS); (2) extinction learning – if the CS is presented repeatedly in absence of the US, the conditioned response gradually diminishes; (3) operant conditioning – behavior can be modified through its association with the consequences, such as addition or removal of rewards or aversive stimuli.

*Delusion formation.* Associative learning informed models propose that individual *neurocognitive vulnerability* (e.g., perceptual and semantic processing Rossell, Batty, & Hughes, 2010; Vinogradov, King, & Huberman, 1992 or attentional capacities, Roy, 2017), or *aberrant neurotransmission* (e.g., dopaminergic, Corlett et al., 2007) increase the likelihood of forming *unusual associations* through classical conditioning. They also hypothesize that *perceptual processing difficulties* (e.g., sensory gating difficulties, Vinogradov, King, & Huberman, 1992), *deficits in conditioned inattention* (Roy, 2017), or *inappropriate mismatch signals* (Corlett et al., 2007) cause a person to *pay attention to irrelevant stimuli*, which also facilitates the formation of unusual associations.

*Delusion maintenance.* The hypotheses on delusion maintenance vary. Some have proposed that initially incidental associations are **reinforced** through operant conditioning, as **feelings of relief or clarity** strengthen these beliefs until they become increasingly rigid and **shape the way further information is processed** (Rossell, Batty, & Hughes, 2010; Vinogradov, King, & Huberman, 1992). Others highlight the amplifying role of fear-driven **avoidance behaviors** that prevent **corrective experiences** as the cues that were misinterpreted to signal threat continue to serve as a warning signal (Moutoussis, Williams, Dayan, & Bentall, 2007). Corlett, Krystal, Taylor, and Fletcher (2009) proposed that **deficits in extinction learning** may underlie delusion maintenance. They drew on the internal reinforcement hypothesis by Eisenhard and Menzel (2007) according to which the presentation of a CS reminds the organism of the US. This reminder is thought to trigger **reconsolidation** of the CS–US association, a process in which recalled memories become labile, integrate new information, and are then restabilized. This process is assumed to compete with extinction learning, in which a new CS–no US association is formed and consolidated. Corlett, Krystal, Taylor, and Fletcher (2009) argued that delusions persist due to inappropriate activity in the midbrain ‘reminder system’ in which aberrant prediction errors might re-evoke the representation of the delusion without disconfirming it. This imbalance would favor reconsolidation over extinction learning, thereby strengthening the delusion rather than extinguishing it.

#### Neurobiological perspectives

Neurobiological perspectives emphasize the role of neuronal alterations in frontotemporal brain regions stemming from *genetic predispositions, neurodevelopmental hazards, psychosocial adversity, and substance use.* These alterations are proposed to disrupt neurodevelopment affecting neurotransmission systems in specific brain regions (the emphasis being on dopamine, but attention is also given to glutamate, norepinephrine, and serotonin), prefrontal cortex monitoring mechanisms, and balance of excitatory and inhibitory mechanisms that then impact perception, learning, and memory. Thus, many neurobiological models are tightly connected with Bayesian and associative learning accounts.

*Delusion formation.* A *dopamine dysregulation* is assumed to lead to the *attribution of aberrant salience to usually irrelevant stimuli* (Howes & Kapur, 2009; Howes & Murray, 2014; Kapur, 2003; Spitzer, 1995), or to an *inappropriate attribution of certainty to sensory information* (Broyd, Balzan, Woodward, & Allen, 2017). The experience of aberrant salience is thought to then lead to the formation of a delusional belief. Some models posit that the *alterations of frontotemporal brain regions* contribute to the altered evaluation of aberrant salience (Poletti & Sambataro, 2013) or other altered perceptions (Carr, 2010). A Bayesian-informed neurobiological model (Denève & Jardri, 2016; Jardri & Denève, 2013) posits that an *imbalance of excitatory and inhibitory (E/I) mechanisms in a hierarchical neural network* causes a pathological form of causal interference in which bottom-up and top-down predictions are reverberated. This is thought to result in the predictions gaining precision – which manifests in erroneous perceptions and beliefs. Recently, it has been proposed that the E/I imbalance leads to abnormal neuronal representations (i.e. shallow attractor dynamics and disrupted generative replay) which manifest as maladaptive beliefs (Nour et al., 2024). Neurobiologically informed two-factor accounts (Coltheart, 2007; Coltheart, Menzies, & Sutton, 2010; Young, 2011) have been proposed for specific types of delusions (e.g., the Capgras delusion, i.e. the belief that a significant other is replaced by an imposter). They are assumed to be driven by a specific neuropsychological impairment (first factor, e.g., a disconnected face recognition system from the autonomic nervous system), triggering a delusional interpretation to explain the (constant) violation(s) of expectation this produces (second factor).

*Delusion maintenance.* Aberrant salience theories do not offer a unique neurobiological account of belief persistence, though some incorporate perspectives from other models (e.g., Bayesian models with the notion that the delusion then serves as a guiding cognitive scheme (or prior) for further thoughts and actions, Jardri & Denève, 2013; Kapur, 2003; see also extended two-factor account by Young, 2011). The two-factor model by Coltheart et al. posits that **damage to lateral regions of right frontal cortex** involves ‘**a failure of the system … to consider new evidence … to revise the current belief’** (2010, p. 282). Spitzer (1995) hypothesized that the longer delusions persist, the more they become **incorporated in the cortical maps** encoding representations of events. Building on research showing **anxiety to favor habitual response patterns and impair neuroplasticity**, he argues that even if the neuromodulatory dysfunctions that led to the delusion (via aberrant salience) have ceased to exist, ‘the “normal” anxiety that accompanies any major changes in one’s world view may be enough to hinder the re-organization of cortical representations’ (p. 101).

#### Vulnerability–stress informed cognitive–behavioral perspectives

The core assumption of vulnerability–stress models is that symptoms result from interaction between predispositional vulnerability and external stress. The vulnerability is explained on different levels, including genetic, biological, or psychological. Here, we focus on models that highlight either cognitive vulnerability in form of biased information processing or emotional vulnerability in form of maladaptive schemas and emotions, or both.

*Delusion formation.* A core idea of these models is that delusional beliefs result from attempts to explain *anomalous, aversive external or internal states triggered by stressors.* The proposed stressors involve adverse childhood experiences and other adverse life events, daily hassles, or sleep dysfunction (e.g., Freeman & Garety, 2014; Garety et al., 2001; Kesting & Lincoln, 2013; Morrison, 2001; Preti & Cella, 2010). This search for an explanation is assumed to be influenced by a cognitive vulnerability in form of *‘biased’ information processing*, such as *selective attention to threat* (e.g., Beck, Rector, Stolar, & Grant, 2009; Bentall, 1994; Blackwood, Howard, Bentall, & Murray, 2001; Salvatore et al., 2012) or a *tendency to jump-to-conclusions* (e.g., Garety et al., 2001; 2007; see Blackwood, Howard, Bentall, & Murray, 2001; Braun & Suffren, 2011 for neuropsychologically informed accounts of information processing deficits), or related biases leading to liberal acceptance of potentially false assumptions (Bronstein et al., 2019; Harrison, Shou, & Christensen, 2021; Moritz et al., 2017). Along this vein, some models build on dual-process theories (Kahneman, 2012) and suggest that *deficits in the mobilization of neurocognitive resources* that support analytic reasoning render the selection of implausible explanations more likely (Bronstein et al., 2019; Speechley & Ngan, 2008; Ward & Garety, 2019). Similarly, the erotetic theory of delusions postulates that people with delusions fail to endogenously generate questions (Parrott & Koralus, 2015).

Some of these models also consider an independent emotional vulnerability in the form of *negative schemas about the self and others*, along with their associated emotions, proposing that such beliefs and emotions shape the appraisal of anomalous or ambiguous experiences (e.g., Freeman et al., 2002; Freeman & Garety, 2014; Jorovat, Twumasi, Mechelli, & Georgiades, 2025; Kesting & Lincoln, 2013; Preti & Cella, 2010; van der Gaag, 2006).

*Delusion maintenance.* Models that focus on cognitive vulnerability generally assume that the same type of reasoning relevant to delusion formation also accounts for delusion maintenance. Some hypothesize additional biases that come into play once a hypothesized explanation is judged as valid: the **‘bias-against-disconfirmatory evidence’** (BADE), where people tend to discard disconfirming evidence altogether, or the **‘confirmation bias’** that describes selectively looking for evidence supporting one’s hypothesis (Moritz et al., 2017).

Models that also emphasize emotional vulnerability point to the effect that either the delusion or its consequences have on emotions and distressing beliefs. For instance, it has repeatedly been suggested that coming up with an explanation, independent of its content, provides initial **relief** (‘insight relief’) or satisfaction and thereby **reinforces the delusional belief** (Freeman et al., 2002; Roberts, 1991). Some suggest that the content of the delusional belief (e.g., paranoid or grandiose) is **negatively reinforced by attenuation of self-doubt or emotional pain** associated with alternative beliefs that pose a threat to the persons’ already low self-esteem (Kesting & Lincoln, 2013). Others propose that **avoidance and safety behaviors** prevent corrective experiences and thereby contribute to belief maintenance (Beck, Rector, Stolar, & Grant, 2009; Freeman, 2016; Freeman et al., 2002; Morrison, 2001; Newman-Taylor et al., 2020).

Finally, some models suggest additional cognitive processes relevant to maintenance: These include the role of **maladaptive metacognitive processes**, such as positive and negative beliefs about one’s beliefs (e.g., their controllability, Morrison, 2001; van der Gaag, 2006), excessive worry or rumination (Freeman, 2016; Isham et al., 2021), the **reactivation of negative schemas about the self and others** that sustain vulnerability beliefs and negative affect (Jorovat, Twumasi, Mechelli, & Georgiades, 2025; Kesting & Lincoln, 2013), and the **relative absence of competing beliefs about safety** that could constrain or override the threat belief (Freeman, Isham, & Waite, 2025).

#### Motivational perspectives

This category summarizes perspectives that emphasize a motive for developing or maintaining a delusion or emphasize a functional aspect. In many cases this is proposed as an additional factor alongside other explanations (e.g., McKay, Langdon, & Coltheart, 2007; Rigoli, Martinelli, & Pezzulo, 2021; Westermann, Gantenbein, Caspar, & Cavelti, 2018). These perspectives build on psychodynamic theories (e.g., Garrett, 2025), theories on the structure of motives (e.g., plan analysis, Caspar, 2011), philosophy (Ritunnano & Bortolotti, 2022), or come from a utility-oriented perspective and tend to view both delusion formation *and* maintenance as a motivated process.

*Delusion formation.* A core assumption is that delusions arise as a way of *dealing with an aversive state* that leaves the person feeling vulnerable, such as a threat of loss of control (Melges & Freeman, 1975), or a threat to self-esteem (Bentall et al., 2001), or anxiety arising from ambiguity and uncertainty (Garrett, 2025; Houran & Lange, 2004). All theories converge on the idea that the delusional experience serves to reduce the *aversive state.* For instance, preserving self-esteem by attributing the causes of current state to external factors (Bentall et al., 2001).

*Delusion maintenance.* The same assumption is made for delusion maintenance. The core postulate here is that maintaining delusional attributions **serves to satisfy psychological needs**, such as **(re)gaining control** (Melges & Freeman, 1975), or achieving a **sense of orientation, coherence, directedness, and belonging** (Ritunnano & Bortolotti, 2022; Westermann, Gantenbein, Caspar, & Cavelti, 2018), or **reducing negative affect** by externalizing overwhelming affects into coherent narratives (Garrett, 2025). It is assumed, for instance, that to maintain a sense of rightness and control, individuals will be motivated to actively seek evidence supporting their delusion (Melges & Freeman, 1975). Some models also describe self-maintaining cycles. For example, persecutory delusions have been proposed to be maintained by the motivation to resist others influence, which increases the chance of **actual rejection from others** (Melges & Freeman, 1975). In addition, delusional beliefs have been postulated to become integrated into **personal identity**, further strengthening the motivation to maintain them (Isham et al., 2021; Melges & Freeman, 1975). Rigoli, Martinelli, and Pezzulo (2021) postulate that some types of delusional beliefs are maintained due to **cost–benefit calculations** (e.g., if the cost of incorrect rejection of a persecutory belief is deemed higher than the cost of incorrectly maintaining it).

#### Social psychological perspectives

Model perspectives from social psychology broadly build on self-categorization (Turner et al., 1987) and social identity theories (Tajfel & Turner, 1979).

*Delusion formation.* These models posit that individuals’ memberships in social groups are integrated into their self-concepts and that *social exclusion or interpersonal adversity* threaten a person’s self-concept and perceived control, creating a basis for interpersonal mistrust that – in interaction with *deficits in social perception and social cognition* – increase the likelihood of misinterpretations and give rise to paranoid beliefs (Fuchs, 1993; Green & Phillips, 2004; Greenaway, Haslam, & Bingley, 2019; Houseman, 1990; Raihani & Bell, 2019).

*Delusion maintenance.* Continued **lack of corrective feedback** by interaction partners and threat avoidance is hypothesized to maintain and exacerbate the beliefs. Along a similar vein, it has also been suggested that paranoid beliefs are strengthened by mutual misunderstanding between interaction partners (Hajdúk, Sasson, Park, & Pinkham, 2024).

## Discussion

### Critical discussion of core postulates

We begin the discussion by summarizing and critically examining three postulates on delusion maintenance that are highlighted in several of the models.

The first relates to operant learning mechanisms and (relatedly) motivational factors. Although not consistently framed in learning terms, most of the models include a mechanistic role of operant learning. A recurring idea is that in a situation characterized by high uncertainty, a delusional explanation is *positively* reinforced by relief or feelings of control and/or *negatively* reinforced by a short-term reduction of uncertainty, emotional distress, or self-doubt. This perspective aligns well with motivational theories pointing to the role of threatened psychological needs (e.g., self-worth, control) that both shape the content of delusions and provide motivation for maintaining them. While this theory is compelling, it needs noting that delusions also tend to have extremely negative consequences, including short-term distress (Freeman, Garety, & Kuipers, 2001; Krkovic, Clamor, Schlier, & Lincoln, 2020) and severely impaired relationships, isolation, and loss of work (Switaj et al., 2012). It may seem counterintuitive that a person would pay such a high price for a short-term sense of control. One could argue, however, that the long-term consequences of a delusion are difficult to anticipate at delusion onset and there is ongoing discussion about whether the costs of giving up a delusional belief may be even higher than the costs of maintaining it (Bortolotti, 2023; Fineberg & Corlett, 2016).

Another frequent assumption is that avoidance behavior (e.g., social withdrawal as a consequence of threat beliefs) leaves the belief unchallenged, thus increasing its probability to persist. This assumption draws on the learning mechanisms postulated to be relevant to the maintenance of anxiety disorders (Craske, Hermans, & Vervliet, 2018) and might struggle to account for delusions that arise in the absence of anxiety or arousal (e.g., grandiosity). It can also not account for delusion maintenance in patients who do not withdraw socially and therefore – in theory – continue to be exposed to disconfirming evidence.

Another postulate highlighted in several perspectives, particularly the Bayesian one, is that the high precision of the delusional beliefs, once formed, influences the way that further sensory input is processed which makes it increasingly difficult to perceive (and integrate) contradicting evidence. Other accounts have made related points using different levels of explanation. For example, the assumption that the longer the beliefs are held the more they are incorporated in the higher-order cortical maps (Spitzer, 1995) also implies that this will influence the way new input is perceived. Although the basic idea that the delusion shapes the way that further input is processed is theoretically compelling and Bayesian models have been developed in close alliance with neurobiological brain models (e.g., Denève & Jardri, 2016; Goodwin et al., 2026), the field would benefit from more explicit specification of concrete computational and neurocognitive mechanisms that implement this influence (e.g., which levels of the hierarchy are affected, how precision is altered, and under which boundary conditions), particularly with respect to longer term delusion maintenance. Another problem with this perspective is that it fails to explain why some people develop strongly held beliefs that become immune to updating while others do not. Moreover, given the assumed impact of top-down beliefs on low-level perceptions, why does momentary insight into the erroneous nature of a delusional belief (i.e. a change of a higher-level belief), as has been described in Capgras delusion (Coltheart, Menzies, & Sutton, 2010), fail to induce the perceptual shifts necessary for stable belief revision? Finally, if one assumes a failure in the weighting mechanisms, it needs to be clarified why this should only occur at specific levels of the hierarchy (or even be of opposite nature at other levels) and why it does not result in a wider array of distorted beliefs.

As a third recurring mechanism, several vulnerability indicators are hypothesized to be relevant to both belief formation and belief maintenance. Among these are frontotemporal alterations that some theories postulate to underlie different types of (neuro)-cognitive processing deficits that play a role in delusion maintenance (e.g., impaired precision weighting of priors or reduced access to neurocognitive resources). Other theories propose an extreme form of a universal information processing bias (e.g., selective attention, jumping-to-conclusions) as a prerequisite for selecting a delusional explanation. Because the jumping-to-conclusions bias does not offer a compelling explanation for delusion maintenance some models include specific ‘maintenance biases’ (such as BADE). The attempt to derive ideas on delusion maintenance from failed extinction learning (Corlett, Krystal, Taylor, & Fletcher, 2009) also falls into this category as it assumes a deficit in updating a previously learnt association. This particular account is complicated by the fact that the conditions under which one would induce reconsolidation over extinction have remained unclear (Schroyens, Beckers, & Luyten, 2023), with some even suggesting that reconsolidation can in fact promote extinction of a fear association. Finally, given that even mentally healthy humans tend to stick to beliefs under certain circumstances (Oeberst & Imhoff, 2023), it appears unlikely that a pronounced neuro-cognitive deficit is a necessary precondition for belief maintenance.

Taken together, while each of these recurring postulates is compelling, they all have caveats and none of them on their own provides a fully convincing account of delusion maintenance.

### Critical discussion of the models’ evidence base

Another limitation of the existing models is the lack of empirical research fully supporting any specific model. Most of the factors proposed to be relevant have been found to correlate with delusions, individually. For example, numerous studies link delusions to aberrant associative learning (e.g., Louzolo et al., 2022; Rossi-Goldthorpe et al., 2024), such as showing recall of extinction learning to be impaired in individuals with delusions (Holt et al., 2012). Delusions also show clear associations with motivationally relevant factors such as low self-esteem (e.g., Jorovat, Twumasi, Mechelli, & Georgiades, 2025; Kesting & Lincoln, 2013) or increased anxiety, avoidance, and safety behavior (e.g., Bennetts, Stopa, & Newman-Taylor, 2021; Denecke, Strakeljahn, Bott, & Lincoln, 2025; Freeman, Taylor, Molodynski, & Waite, 2019b). There is also growing empirical support for the idea that (higher-order) prior beliefs can shape perceptual inference and evidence integration in delusion proneness and psychosis (for reviews see: Goodwin et al., 2026; Katthagen, Fromm, Wieland, & Schlagenhauf, 2022). As such, most factors proposed to be relevant in the models are all evidence based to a certain extent. Nevertheless there is a lack of empirical research fully supporting the causal postulates of any of the given models. This is particularly clear for models that assign a central maintaining role to a deficit (or stable bias). These models would need to show that the proposed deficit is not only strongly correlated with and temporally predictive of delusions, but also that reducing the deficit has a pronounced effect on delusion maintenance. To our knowledge, this has yet to be shown for any of the proposed deficits or biases and even correlative evidence has not been compelling for the biases proposed for maintenance specifically, such as the BADE (Samson et al., 2024). Similarly, to back up models that postulate a mechanistic role of operant or associative learning, studies need to directly address this mechanistic assumption, such as by testing whether a delusional explanation has short-term benefits on self-esteem or whether avoidance behavior predicts increased threat beliefs. Single attempts to demonstrate these mechanisms (e.g., Lincoln, Stahnke, & Moritz, 2014) and the effectiveness of interventions that place a strong emphasis on reducing safety behaviors (Freeman et al., 2021; Pot-Kolder et al., 2018) provide indirect evidence, but the required direct mechanistic research is scarce.

In addition, the combined predictive values and interactions proposed in the more complex multifactorial models have remained largely untested. Freeman and his colleagues (Loe et al., 2023; Freeman et al., 2005) tested numerous predictors derived from their models cross-sectionally in healthy samples. They found several of the proposed maintenance factors, such as defense behaviors and avoidance, to be among the strongest predictors of paranoid delusion severity. While these studies underline the potential relevance of the proposed factors, they do not map directly onto a specific model and the amount of unexplained variance indicates that relevant factors are still missing.

To sum up, although the theories on delusion maintenance provide numerous interesting hypotheses, none of them on their own are sufficiently compelling or empirically sufficiently backed up to provide a complete and satisfactory explanation of delusion maintenance.

### An integrated framework

**Figure 2.**
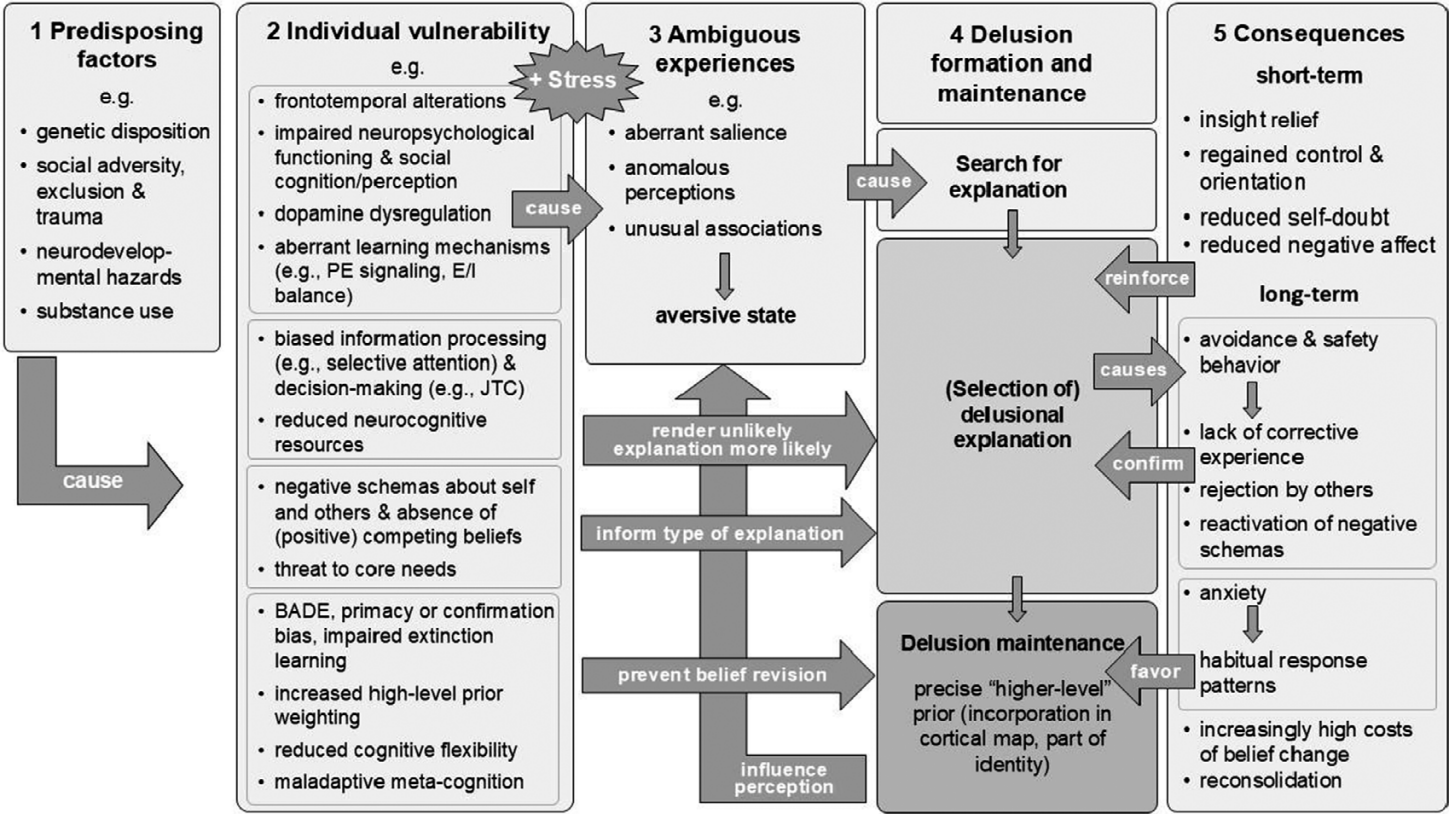
Summary and integration of the core factors proposed for delusion formation and maintenance. *Note*: The integrated model includes (1) risk factors, (2) the vulnerability that results from the risk factors which includes factors predominantly relevant to formation (top 2 subsections), maintenance (bottom subsection), or both; (3, 4) the proposed mechanism of formation (i.e. search for explanation of ambiguous subjective experiences) and (5) the short- and long-term consequences postulated to be responsible for belief maintenance and chronification (4) along with feedback loops. BADE, bias against disconfirmatory evidence; E/I, excitatory/inhibitory; JTC, jumping to conclusions; PE, prediction error.

One way of gaining a more comprehensive understanding of delusion maintenance is to integrate the postulates from the individual models into a unifying model. Combining models is likely to yield a higher explanatory value than any specific model on its own. By highlighting potential interactions and temporal successions, a unified model can also help to stimulate further research and approaches for intervention. To this end, we subdivided the main postulated factors and mechanisms into five stages that we visualize in Figure 2.

As all models conceptualize delusions as an attempt to explain some kind of ambiguity (albeit using different terminology and stressing different factors and mechanisms that account for this experience), this mechanism is a central element of the integrated model (see stage 3, Figure 2 middle part). Besides ongoing stressors, many of the models specify risk factors that leave people vulnerable both neurobiologically and psychologically (stages 1 and 2, Figure 2, left-hand side). The proposed vulnerability factors can be subdivided into four categories. The first category contains vulnerability specified at a biological and neurocognitive level proposed to both account for these ambiguous experiences (e.g., dopamine dysregulation by affecting the perceived salience of stimuli) and give rise to some of the other vulnerability factors (e.g., impaired information processing, cognitive flexibility). The second category is postulated to increase the likelihood of selecting a delusional explanation (e.g., biased information processing, such as jumping to conclusions, will increase the probability of selecting an unlikely explanation). The third category is assumed to influence which type of explanation is selected (e.g., people with generalized negative-other schemas are more likely to select a paranoid explanation). The fourth category describes a type of vulnerability that renders it less likely that, once selected, a delusional explanation will be revised (e.g., the metacognitive belief that it is safer to be paranoid could reduce the motivation to take disconfirming evidence into account). Over and above vulnerability, the models describe maintenance mechanisms that arise from the delusional explanation as such (stage 5, Figure 2, right-hand side). One involves operant learning mechanisms that arise via the consequences of the beliefs. Immediate short-term consequences (e.g., insight relief) are assumed to reinforce the delusional explanation (e.g., the relief that comes with the reduced feeling of confusion due to having come up with an explanation). Operant learning is also implicated in the proposed effect of longer term consequences of the behavior that follows delusions (e.g., if acting on the delusion leads to rejection by others, this will further confirm the paranoid content of a delusion, possibly by reactivating preexisting negative schemas about others). Other proposed factors are assumed to impact negatively on belief revision as such. For instance, anxiety and distress resulting from delusions or their consequences are proposed to favor habitual response patterns and thus decrease the likelihood of belief revision. In addition, with respect to motivational perspectives, it can be speculated that the costs of belief revision increase over time (e.g., the longer a person has held the belief and acted on it the more shameful it becomes to discard it). Finally, the Bayesian-informed assumption - that delusions take on the form of top-down beliefs that influence the way that further sensory input is processed which makes it increasingly difficult to perceive (and integrate) contradicting evidence - is depicted by a feedback loop from delusions to perceptual experiences.

### Clinical implications

So far, most interventions for delusions have focused on one or few of the proposed mechanisms. Given that they tend to produce only small effects (e.g., CBTp: Mehl, Werner, & Lincoln, 2015; reasoning focused approaches: Garety et al., 2021; Penney et al., 2022), it would be interesting to explore whether they can be improved by taking additional mechanisms into account. For instance, the effect of cognitive approaches and reasoning trainings might be increased if they succeeded in reducing the reinforcing consequences of delusions by finding alternative ways to increase a sense of control, reduce anxiety, or satisfy core psychological needs. Promising recent developments that focus on reducing avoidance behavior (Freeman et al., 2019a; Pot-Kolder et al., 2018) might be even more successful if they consider that delusional beliefs could be shaping the way the environment is perceived. Belief updating could be facilitated by making sure the expectations are formulated in a testable manner, the contradictory evidence is sufficiently clear, and violations of expectations are processed (Schemer, Körfer, & Glombiewski, 2020). Another option is to attempt to create a counterweight to the delusional (paranoid) belief by forming a new safety belief that can coexist with the person’s existing belief (Freeman, Isham, & Waite, 2025). Once established, this new belief might also shape the way new information is processed, making it easier to learn from evidence that contradicts the delusion.

Although it is possible that multiple pathways lead to the same outcome and that therefore targeting a single factor can be sufficient for some patients, it is yet unknown which factors play a role for whom. The combined framework could be used to inform an individualized assessment of risk and vulnerability factors, triggering experiences, beliefs, and their consequences as a basis for personalized intervention. One way to plan this could be to use functional analysis as has also been suggested for negative symptoms (Lincoln et al., 2017).

### Directions for future research

One direction for future research is to test whether combining the factors across models explains a higher amount of variance than single models or perspectives. It also needs testing whether the different factors are equally relevant to delusions or whether their predictive value differs between diagnostic categories or at different levels of severity. Also, further research is needed to refine the models. Some putative factors are not well specified. These include the role of neurobiology in delusion maintenance and the role of ongoing psychosocial adversity (Jaya & Lincoln, 2016; Pries et al., 2020). Also, none of the models provide a satisfactory mechanistic account of what happens in the belief-updating process (or non-updating process) when a delusional belief is challenged by conflicting evidence. Also, the long-term mechanisms require further specification. In our view, even the integrated model fails to sufficiently explain the development of systematized delusions or the re-emergence of the same delusional content after phases of remission (Grunfeld et al., 2024).

Although many models were informed by basic psychological perspectives, most now lag behind the contemporaneous development in the fields of their respective perspectives. There have been several psychological accounts attempting to explain belief persistence in healthy populations (Pinquart et al., 2021). To move our understanding of delusion maintenance forward, it therefore appears promising to search for further input from relevant fields of psychology that can provide a basis for a mechanistically oriented refinement of the existing models. Recent developments in associative learning research outline putative mechanisms that support belief maintenance despite contradictory evidence. These include shifting attention to contextual cues (Darby & Pearce, 1995; Kruschke, 2001) or attributing the unexpected outcomes to cues with uncertain causal status (Chow, Lee, & Lovibond, 2024; Spicer, Mitchell, Wills, & Jones, 2020). Similarly, recent developments in Bayesian frameworks have put forward accounts of how the brain accommodates contradictory evidence without changing the core of a belief, such as by forming additional beliefs (Gershman, 2019). In addition, work on delusions arising in the context of focal neurological conditions (e.g., epilepsy or hippocampal lesions) may provide important complementary constraints on the mechanisms underlying belief formation and maintenance and could help to further refine and test these accounts. Finally, the explanatory value of the motivational perspectives and their testability might be further improved by building on recent utility-informed perspectives. For example, Sharot, Rollwage, Sunstein, and Fleming (2023) frame belief formation and maintenance as a multiattribute value-based decision-making problem, where the aim is to hold the belief with the highest value – which is not necessarily the accurate belief. These new developments lend themselves well as a basis for deriving novel hypotheses on the cognitive and computational mechanisms of delusion maintenance (Lincoln, Romberg, Torrents-Rodas, & Bott, 2026).

## Supporting information

10.1017/S0033291726103705.sm001 Lincoln et al. supplementary material Lincoln et al. supplementary material

## Supplementary material

The supplementary material for this article can be found at http://doi.org/10.1017/S0033291726103705.
