# Supplementary material for: A review and integration of models on delusion maintenance
Source: Psychol Med. 2026 May 5;56:e127. doi: 10.1017/S0033291726103705 (PMC13161818; doi:10.1017/S0033291726103705)
Supplement: Lincoln et al. supplementary material [file S0033291726103705sup001.docx]

**Supplementary Table**

*Included models of delusion maintenance and their core postulates.*

| Publication | Core perspective | Article type | Core postulate on delusion maintenance |
| --- | --- | --- | --- |
| Adams et al. (2013) | Bayesian inference | Theoretical model | Aberrant precision-weighting yields unstable low-level priors; once adopted, high-level delusional priors guide perception and explain away disconfirming evidence |
| Ashinoff et al. (2022) | Bayesian inference | Selective review (computational psychiatry perspective) | Proposes that delusional rigidity can arise from overweighted priors and reduced updating to new evidence (primacy/‘stickiness’), yielding persistent high-certainty beliefs |
| Beck et al. (2009) | Cognitive-behavioral | Theoretical model | Threat schemas and safety behaviors prevent disconfirmation; biased appraisal maintains delusional meanings |
| Bentall (1994) | Cognitive-behavioral | Theoretical model | Cognitive-bias account: biased attribution/interpretation processes protect beliefs from disconfirming evidence |
| Bentall et al. (2001) | Motivational | Theoretical model | Persecutory delusions can protect self-esteem; motivated attributions and defensive processing help maintain beliefs |
| Blackwood et al. (2001) | Cognitive-behavioral | Theoretical model | Neuropsychologically informed cognitive model: abnormal threat perception and reasoning biases sustain persecutory beliefs |
| Bronstein et al. (2019) | Cognitive-behavioral | Theoretical model | Dual-process/conflict model: reduced analytic reasoning and conflict processing limits belief correction, supporting persistence |
| Coltheart et al. (2010) | Neurobiological | Theoretical model | Abductive-inference account: right frontal damage yields failure to consider new evidence and revise the delusional belief |
| Corlett et al. (2007) | Associative learning theory | Theoretical model | Aberrant prediction errors drive maladaptive associations; reinforcement of these associations and failure to correct them support persistence |
| Corlett et al. (2009) | Associative learning theory | Theoretical model | Failed extinction vs. reconsolidation: prediction errors repeatedly reconsolidate delusional associations rather than extinguish them |
| Corlett et al. (2016) | Bayesian inference | Theoretical model | Aberrant prediction-error signaling sustains belief-updating dysfunction and promotes persistence by repeatedly reinstating the delusional explanatory model |
| Denève & Jardri (2016) | Bayesian inference | Computational/theoretical model | Circular inference (reverberating bottom-up/top-down signals) increases certainty and fosters self-reinforcing beliefs resistant to contradictory evidence |
| Diaconescu et al. (2019) | Bayesian inference | Theoretical model | Distinguishes formation vs. consolidation: after adoption, an “extraordinary” higher-order belief exerts strong top-down control, stabilizing the delusion |
| Feeney et al. (2017) | Bayesian inference | Theoretical model | Delusions reduce uncertainty; persistence follows when the delusional hypothesis continues to minimize expected uncertainty/costs better than alternatives |
| Fineberg & Corlett (2016) | Bayesian inference | Theoretical model | Learning/memory account: maladaptive reconsolidation and prediction-error processing “pins” delusional beliefs, limiting revision |
| Fletcher & Frith (2009) | Bayesian inference | Computational/theoretical model | Predictive-coding account: faulty precision and prediction errors bias inference; once established, priors dominate perception, reducing impact of counterevidence |
| Freeman & Garety (2014) | Cognitive-behavioral | Theoretical model | Cognitive treatment model: persistent paranoia maintained by biases, negative affect, and avoidance/safety behaviors |
| Freeman et al. (2002) | Cognitive-behavioral | Theoretical model | Cognitive model of persecutory delusions: safety behaviors, threat anticipation, and reasoning biases maintain beliefs |
| Freeman et al. (2025) | Cognitive-behavioral | Theoretical clinical model (treatment-oriented synthesis) | Persecutory delusions persist through interacting maintaining processes (e.g., worry, threat monitoring, avoidance/safety behaviours, negative self-beliefs, anomalous experiences, reduced access to alternative explanations, sleep/activity disruption, environmental adversity), and the relative absence of competing safety beliefs |
| Friston (2008) | Bayesian inference | Theoretical model | Hierarchical predictive processing: belief persistence arises when higher-level priors retain high precision and constrain lower-level evidence integration |
| Friston et al. (2016) | Bayesian inference | Theoretical model | Disconnection/precision-gain account: synaptic gain abnormalities impair updating and promote overly stable priors that resist revision |
| Fuchs (1993) | Social | Theoretical model | Lack of corrective feedback and social misattunement maintain persecutory interpretations, especially under sensory impairment |
| Garety et al. (2001) | Cognitive-behavioral | Theoretical model | Cognitive model of positive symptoms: appraisal of anomalous experiences plus reasoning biases and safety behaviors maintain delusions |
| Garety et al. (2007) | Cognitive-behavioral | Theoretical model | Theoretical paper: biased reasoning and threat appraisals interact with neurobiology; maintenance via persistent biases and coping strategies |
| Garrett (2025) | Motivational | Theoretical integration (CBTp and neuropsychoanalytic perspectives) | Suggests delusional narratives can be maintained because they regulate overwhelming affect after trauma by providing coherent, externalised meanings that stabilize emotional states |
| Green & Phillips (2004) | Social | Theoretical model | Social-threat perception account: ongoing interpersonal threat cues and misinterpretation maintain paranoia |
| Greenaway et al. (2019) | Social | Theoretical model | Social identity model of paranoia: threatened group-based self-concept and mistrust promote persistence of paranoid beliefs |
| Griffin & Fletcher (2017) | Bayesian inference | Theoretical model | Source-monitoring/predictive-processing failures maintain delusions by misattributing internally generated information as external confirmation |
| Hajdúk et al. (2024) | Social | Theoretical model | Misattunement account: mutual misunderstanding in interaction strengthens paranoia through escalating feedback cycles |
| Harding et al. (2024) | Bayesian inference | Computational/theoretical model | Comprehensive predictive-coding model: delusions persist via feedback loops between priors, attention, and action that continually protect the belief |
| Harrison et al. (2021) | Cognitive-behavioral | Theoretical model | Correlation-detection dysregulation: spurious pattern detection persists and resists correction, stabilizing delusional interpretations |
| Hemsley & Garety (1986) | Bayesian inference | Theoretical synthesis | Bayesian analysis: atypical weighting of evidence vs. expectations proposes that reduced use of disconfirmatory evidence and stability of delusional hypotheses |
| Houseman (1990) | Social | Theoretical model | Biopsychosocial model: interpersonal adversity and reinforced mistrust sustain paranoid beliefs |
| Howes & Kapur (2009) | Neurobiological | Theoretical model | Dopamine dysregulation and aberrant salience: once a delusional explanation forms, it becomes a guiding schema sustaining interpretation of salience |
| Howes & Murray (2014) | Neurobiological | Theoretical model | Integrated sociodevelopmental-cognitive model: ongoing dopaminergic/socio-cognitive dysregulation and stress maintain symptoms including delusions |
| Jorovat et al. (2025) | Cognitive-behavioral | Systematic review and meta-analysis (model synthesis) | Links negative core beliefs (self/others) to persecutory delusions and suggests persistence via reactivation of these beliefs and associated negative affect shaping threat appraisals |
| Kapur (2003) | Neurobiological | Theoretical framework | Aberrant salience framework: delusions persist as a cognitive explanation that organizes ongoing salience experiences |
| Kesting & Lincoln (2013) | Cognitive-behavioral | Theoretical model | Self-esteem/self-schema account: delusions can reduce self-doubt and are maintained via negative reinforcement and schema-consistent processing |
| Melges & Freeman (1975) | Motivational | Theoretical model | Cybernetic model: delusions maintain control/orientation; active evidence-seeking and interpersonal cycles reinforce beliefs |
| Miyazono & McKay (2019) | Bayesian inference | Theoretical model | Hybrid model: interacting deficit and motivational factors maintain delusions by stabilizing belief selection and discouraging updating |
| Moritz et al. (2017) | Cognitive-behavioral | Theoretical model | Two-stage cognitive theory: lowered decision thresholds and confirmation processes maintain false beliefs once adopted |
| Morrison (2001) | Cognitive-behavioral | Theoretical model | Interpretation-of-intrusions model: metacognitive beliefs about intrusions maintain psychotic appraisals and prevent reappraisal |
| Moutoussis et al. (2007) | Associative learning theory | Theoretical model | Conditioned avoidance: fear-driven avoidance prevents corrective learning, maintaining persecutory beliefs |
| Newman-Taylor et al. (2020) | Cognitive-behavioral | Theoretical model | Cannabis-related paranoia model: safety behaviors and threat appraisals maintain paranoia via avoidance and biased interpretation |
| Parrott & Koralus (2015) | Cognitive-behavioral | Theoretical model | Erotetic theory: impaired question-generation reduces critical inquiry, limiting belief revision and sustaining delusions |
| Petrovic & Sterzer (2023) | Bayesian inference | Theoretical model | “Delusion paradox” resolution: high-level priors can become hyper-precise and sculpt perception into conformity, buffering against contradiction |
| Pfuhl (2017) | Bayesian inference | Computational/theoretical model | Task-based Bayesian perspective: altered priors/decision thresholds can keep delusion-like beliefs stable despite disconfirming information |
| Poletti & Sambataro (2013) | Neurobiological | Theoretical framework | Transdiagnostic developmental framework: persistent evaluation/monitoring alterations maintain delusional interpretations once formed |
| Preti & Cella (2010) | Cognitive-behavioral | Theoretical model | Heuristic account: paranoia as a heuristic persists because it is quick, low-cost, and reinforced by perceived threat reduction |
| Raihani & Bell (2019) | Social | Theoretical model | Evolutionary-social account: threat monitoring and strategic mistrust can be self-reinforcing, supporting persistence of paranoia |
| Ritunnano & Bortolotti (2022) | Motivational | Theoretical model | Meaning-based account: delusions can confer meaning and are maintained when they support coherence/identity |
| Roberts (1991) | Cognitive-behavioral | Theoretical model | Meaning/preferred reality account: delusional systems provide meaning and are maintained because alternatives are existentially costly |
| Rossell et al. (2010) | Associative learning theory | Theoretical model | Cognitive-associative model: operant reinforcement (relief/clarity) strengthens unusual associations and biases subsequent information processing |
| Roy (2017) | Associative learning theory | Theoretical model | Conditioning model: attentional/learning abnormalities promote persistent cue–outcome associations that are not extinguished by counterevidence |
| Salvatore et al. (2012) | Cognitive-behavioral | Theoretical model | Vulnerable self/mentalizing model: threat anticipation and biases in understanding others perpetuate persecutory interpretations |
| Speechley & Ngan (2008) | Cognitive-behavioral | Theoretical model | Dual-stream modulation failure: impaired integration and monitoring sustain delusional interpretations over time |
| Spitzer (1995) | Neurobiological | Computational/theoretical model | Neurocomputational approach: delusions become embedded in cortical representations; anxiety and reduced plasticity hinder reorganization and updating |
| van der Gaag (2006) | Cognitive-behavioral | Theoretical model | Remission model: metacognitive and affective processes (and their change) influence persistence vs. remission of delusions |
| Vinogradov et al. (1992) | Associative learning theory | Theoretical model | Associationist/paranoid process: spreading activation and aberrant gating sustain associative networks, stabilizing delusional interpretations |
| Ward & Garety (2019) | Cognitive-behavioral | Review / theoretical synthesis | Fast/slow thinking review: reliance on fast reasoning maintains delusions; limited slow analytic processing prevents updating |
| Westermann et al. (2018) | Motivational | Theoretical model | Need-satisfaction model: delusions are maintained because they satisfy and protect psychological needs |
| Young (2011) | Neurobiological | Neuropsychological theoretical model | Interactionist two-factor extension: continuing anomalous experiences plus impaired belief evaluation/interaction dynamics sustain misidentification beliefs |
| Zubin & Spring (1977) | Cognitive-behavioral | Theoretical framework | Vulnerability-stress framework: persistent stress and vulnerability maintain symptom expression, including delusional beliefs |

*Note*. Publications are listed alphabetically. The column “Best-fitting core perspective” indicates the core perspective used in the manuscript to classify each model.
